# Sugar Content and Nutritional Quality of Child Orientated Ready to Eat Cereals and Yoghurts in the UK and Latin America; Does Food Policy Matter?

**DOI:** 10.3390/nu12030856

**Published:** 2020-03-23

**Authors:** Ada L. Garcia, José D. Ronquillo, Gabriela Morillo-Santander, Claudia V. Mazariegos, Lorena Lopez-Donado, Elisa J. Vargas-Garcia, Louise Curtin, Alison Parrett, Antonina N. Mutoro

**Affiliations:** 1 Human Nutrition, School of Medicine, Dentistry and Nursing, College of Medical, Veterinary and Life Sciences, University of Glasgow, New Lister Building, 10-16 Alexandra Parade, Glasgow G31 2ER, UK; davidronquillo27@gmail.com (J.D.R.); Alison.Parrett@glasgow.ac.uk (A.P.); antoninanamaemba@gmail.com (A.N.M.); 2 School of Nutrition, Faculty of Medicine and Health Sciences, Mariano Galvez University, 3a. Avenida 9-00 zona 2, 01002 Interior Finca El Zapote, Guatemala 01002, Guatemala; clavima1@gmail.com (C.V.M.); ldonado@umg.edu.gt (L.L.-D.); 3 School of Food Science and Nutrition, Department of Health Sciences, Universidad Iberoamericana León, Blvd. Jorge Vértiz Campero 1640, Colonia Cañada de Alfaro, 37238 León, Gto., Mexico; elisa.vargas08@gmail.com

**Keywords:** sugar, food labelling, sugar reduction policy, children, food marketing

## Abstract

Ready to eat breakfast cereals (REBCs) and yoghurts provide important nutrients to children’s diets, but concerns about their high sugar content exist. Food reformulation could contribute to sugar reduction, but policies across countries are not uniform. We aimed to compare the sugar content and nutritional quality of child-orientated REBCs and yoghurts in Latin American countries with the UK. In a cross-sectional study, nutritional information, marketing strategies, and claims were collected from the food labels and packaging of products available in Guatemala, Mexico, Ecuador and the UK. Nutritional quality was assessed using the UK Ofcom Nutrient Profiling System. In total, 262 products were analysed (59% REBCs/41% yoghurts). REBCs in the UK had a lower sugar content (mean ± SD) (24.6 ± 6.4) than products in Ecuador (34.6 ± 10.8; *p* < 0.001), Mexico (32.6 ± 7.6; *p* = 0.001) and Guatemala (31.5 ± 8.3; *p* = 0.001). Across countries, there were no differences in the sugar content of yoghurts. A large proportion (83%) of REBCs and 33% of yoghurts were classified as “less healthy”. In conclusion, the sugar content of REBCs in Latin America is higher than those of the UK, which could be attributed to the UK voluntary sugar reduction programme. Sugar reformulation policies are required in Guatemala, Mexico and Ecuador.

## 1. Introduction

Reducing the sugar content of manufactured foods aimed at children is a public health strategy to promote healthier food environments [1,2]. Recommended actions to implement sugar reduction include policies and regulations aimed at reformulation, reduction in portion size, taxation, front-of pack labelling, restrictions on marketing of child-orientated foods that are high in sugar, price promotion control and/or sugar awareness campaigns [2,3,4]. However, the degree of policy implementation varies across countries. In the UK, sugar reduction in foods has been mainly channelled through taxation of sugar-sweetened beverages and a voluntary sugar reformulation programme. Latin American countries have made some progress on policy, but this is patchy. In Mexico and Ecuador, sugar reduction in foods has been limited to taxation of sugar-sweetened beverages [5,6], whereas countries like Guatemala have not implemented sugar reduction policies aimed at specific food groups. These differences in policy provide an important opportunity to study the potential impact of the voluntary sugar reformulation programme aimed at specific food groups in the UK compared with countries without this policy. 

The UK voluntary sugar reduction programme started in 2017 with a target to reduce total sugar content of nine specific food groups that contribute to high sugar consumption in children by 5% in the first year and to achieve 20% reduction by 2020. Food groups included core foods (e.g., ready to eat breakfast cereals (REBCs) and yoghurts) and non-core foods (e.g., chocolate and confectionary) [7]. 

We focused on REBCs and yoghurts because they are important contributors to children’s nutrient intake in both the UK and Latin America. REBCs have become part of the diet patterns of children in Latin America [8,9], while yoghurt sales are projected to grow [10]. In the UK, cereals are the main sources of micronutrients in children aged 1.5 to 10 years, while yogurts provide 5% of protein and 8% of calcium intake in children aged 1.5 to 10 years [11]. Similar findings are reported for REBCs in Guatemala [12], Mexico [8] and Ecuador [13]. However, there is a well- established body of evidence that consistently highlights concerns about the nutritional quality of REBCs and yoghurts marketed to children, in particular the high sugar content, while other nutrients of concern are fat, sodium, protein and fibre [9,14,15,16,17,18,19,20,21,22,23,24,25,26,27]. In the UK, REBCs and yoghurts contribute to 15% and 16% of free sugar intake in children aged 1.5–10 years, respectively [11]. A systematic review of observational studies found that frequent consumption (>5 servings/week) of REBCs in children was associated with higher total sugar consumption [28]. It has also been a matter of controversy that these products rely heavily on marketing strategies that contradict WHO guidance. Concerns exist around the use of child-friendly imagery on product packaging [29], promotions via TV advertisement [30] and the use of unregulated nutrition [31] or health claims to promote a “healthy halo” image when these foods contain are classified as “less healthy” because of high sugar, salt and low fibre content [19].

We hypothesised that the voluntary sugar reformulation programme in the UK will have an impact on the nutritional quality, particularly sugar content, of REBCs and yoghurts marketed towards children compared with three Latin American countries where there is no food-targeted sugar reduction policy. The aim of the study was to compare the sugar content and nutritional quality of child-orientated REBCs and yoghurts in three Latin American countries with the UK.

## 2. Materials and Methods 

### 2.1. Study Design

A cross-sectional study of REBCs and yoghurts was conducted in a large city in the UK and in three large cities in Latin America: Glasgow, United Kingdom (UK); Guayaquil, Ecuador; Guatemala City, Guatemala; and Merida, Mexico. 

The three largest grocery retailers with the most market share were used for data collection in the UK. In large cities in Latin America, socioeconomic inequalities are largely marked by spatial boundaries that influence supermarket segmentation and the type of products on sale according to income levels (low, middle and high) [32,33,34,35]. Therefore, in Latin America, three large retailers with a significant share of the market representing different income segments (low, middle and high) were selected to maximise product range. We also used the researcher’s local knowledge of the respective cities to inform supermarket selection. 

In the UK, “Tesco” “Asda” and “Aldi” accounted for 50.7% of the market share in 2019 [36,37]. In Guayaquil, supermarkets were selected based on the targeted income level [34], “Supermaxi”, “Mi Comisariato” and “Almacenes TIA” were selected to represent high-, middle- and low-income supermarkets, respectively. These three retailers represented 87% of the market share in Ecuador in 2015 [38]. In Guatemala City, “Walmart”, “La Torre” and “Despensa Familiar” represented high-, middle- and low-income supermarkets, respectively [33]. In Guatemala City, the grocery retailing system is largely dictated by the purchasing capacity of its population, which is influenced by location (zones) [33]. Despensa Familiar was located in zone 18, a locality with high levels of poverty; La Torre was located in Calzada Roosevelt, zone 11, a middle-class zone, and Walmart was located on the outskirts of Guatemala City in zone 15, a wealthy area, on the road to El Salvador. La Torre and Despensa Familiar have 22% of supermarket share in Guatemala City [33]. In Merida, Chedraui Selecto represented high-income, Walmart middle-income, and Soriana low-income supermarkets. These retailers represent approximately 50% of the market share in Mexico [39]. In Merida, Soriana, a low-income supermarket, are found in the vicinity of community dining halls which aim to improve access to food in highly marginalized communities [40]; Walmart was located in the city centre, which is a middle-income neighbourhood, whereas Chedraui Selecto is a specialized “gourmet” supermarket, a trendy development in the grocery business aimed at high-income earners [35]; this was located in the northwest of the city [41].

### 2.2. Inclusion Criteria and Data Collection

REBCs and yoghurts were included if they had cartoon characters (licensed or unlicensed), child-friendly images, puzzles, games, toys and activities or promotions appealing to children, or terms such as ‘children’, ‘child’, ‘kids’, or ‘little/young ones’ and references to ‘lunch box’ or ‘growing up’ on the product packaging. Yogurts included those sold in pots and squeezable bags, yoghurt drinks and fromage frais. Products that were targeted at babies (e.g., cereals recommended from 4 to 12 months in the baby section), adults or without any references to children were excluded from this study. This methodology has previously been used by our group [19].

Using the product label and packaging, the following information was recorded: origin of the product (to define if the product was produced by a local or multinational/international company), serving size and energy and nutritional information for total fat, saturate fat, carbohydrates, protein, sugar, fibre, salt (all based on g/100g of product). The use of health and nutrition claims and child-orientated marketing strategies, such as cartoons, toys and promotions, were also recorded. Health claims are statements about a relationship between food and health [42]. They can either be health claims relating to function, for example, growth, development and functions of the body (immune system, digestion or cognitive development) or risk reduction claims [42]. Nutrient claims are specific to nutrient content (e.g., added micronutrients, low in sugar, high in calcium/fibre). Other claims included statements such as gluten-free, lactose-free or high in protein.

The data were collected between September and December 2018 by two independent researchers in each country. The researchers collected data independently but used the same protocol. Data entry was compared, and if discrepancies existed, data were checked again before consolidation into one database. In the UK, Mexico and Ecuador, all data were collected both by physical visits to stores and via online searches of supermarket websites (UK) or producers’ websites (Mexico and Ecuador). In Guatemala, data were collected in store only. 

### 2.3. Nutrient Profiling Model (NPM)

The nutritional quality of the food products was assessed using the 2011 Ofcom Nutrient Profiling Model (NPM), originally implemented to differentiate between the nutritional quality of foods in order to restrict those advertised to children [43]. This model classifies foods using a scoring system calculated from nutrient and food component information on food labels. The score consists of 7 components, 4 negative (energy, total sugars, saturated fat and sodium) and 3 beneficial (fruit, vegetables and nuts, fibre and protein). The beneficial score is subtracted from the negative score, and a total score is calculated. Foods and non-alcoholic drinks are classified as “less healthy” if foods score 4 or more points, and drinks score one or more points per 100 g, respectively. The NPM classifies foods as “healthy” or “less healthy”; healthy implies a healthful nutrient profile in foods. Chi square tests were used to test associations between health and nutrition claims and the Ofcom NPM. 

### 2.4. Statistical Analysis 

Data are shown using descriptive statistics. Analysis of variance was used to test the difference in nutrient content between Mexico, Guatemala, Ecuador and the UK for nutrients which were normally distributed. Because multiple comparisons were made, the Bonferroni correction test was used to reduce the chance of type 1 error. For non-parametric data, the Kruskal–Wallis test was used to assess differences in nutrient content. IBM Corp. Released 2011. IBM SPSS Statistics for Windows, Version 20.0. Armonk, NY, USA: IBM Corp. was used for data analysis. Significance was accepted at *p* < 0.05. 

## 3. Results

### 3.1. Sample Characteristics

Two hundred and sixty-two REBCs and yoghurt products were identified; one third (32.4%) from the UK, 23.7% from Ecuador, 22.1% from Mexico and the rest from Guatemala. Over half of the products were REBCs (59.1%), and over half (55.3%) were manufactured by multinational companies. The child-orientated marketing strategies used in REBCs and yoghurts in this survey are shown in Table 1. A large proportion of the yoghurts and REBCs used cartoon characters for marketing across all four countries (89.7%). Health- and nutrient-related claims as a form of marketing are also presented. Health (43.5%) and micronutrient (82.4%) claims were more common on products in the UK, whereas nutrient specific claims such as low/no added sugar and low saturated fat were more common in Mexico. 

### 3.2. Ready to Eat Breakfast Cereals (REBCs)

A comparison of the nutrient composition (all shown as g/100 g) of REBCs and yoghurts sold in Latin America (Mexico, Ecuador and Guatemala) and the UK is presented in Table 2 and Table 3, respectively. Compared with products sold in the UK (24.6 ± 6.4, mean ± SD), cereal products from all Latin American countries had significantly higher sugar content (Ecuador (34.6 ± 10.8; *p* < 0.001), Mexico (32.6 ± 7.6; *p* = 0.001) and Guatemala (31.5 ± 8.3; *p* = 0.001)). Similarly, their carbohydrate (median (IQR)) content was also higher, but this difference was only statistically significant for products from Mexico (84.0 (80.0, 86.6), *p* = 0.005) and Ecuador (86.7 (82.5, 86.7), *p* < 0.001). In contrast, the protein content of cereal products was significantly lower in Latin American countries (mean ± SD) (Ecuador (5.7 ± 2.1, *p* = 0.03), Mexico (5.2 ± 2.2, *p* = 0.001) and Guatemala (5.2 ± 1.6, *p* < 0.001)). The saturated fat and salt content were also significantly lower in Ecuador (Table 2). Overall, 83.1% of breakfast cereals were classified as “less healthy”. Compared with products from the UK, products from Mexico, Guatemala and Ecuador were more likely to be classified as “less healthy” (Table 2). 

Overall, local REBCs in the UK had a lower sugar content, mean ± SD (23.5 ± 5.5), than local products in Ecuador (38.2 ± 12.8; *p* < 0.001), Mexico (30.6 ± 9.8; *p* = 0.2) and Guatemala (25.0 ± 10.4; *p* = 0.1), but the difference was only statistically significant in Ecuador. Similarly, the sugar content of multinational products in the UK (25.8 ± 7.37) was lower than that in multinational products sold in Ecuador (31.9 ± 8.3; *p* = 0.06), Mexico (34.0 ± 5.5; *p* = 0.005) and Guatemala (33.1 ± 7.1; *p* = 0.005). 

### 3.3. Yoghurts

For yoghurts, there was no significant difference in sugar content between the UK and the three Latin American countries (Table 3). Compared with the UK, yoghurt products in Guatemala had, on average, higher salt (mean ± SD) (0.56 ± 0.02, *p* < 0.001) and carbohydrate content (median (IQR)) (16.3 (14.0, 19.5) *p* = 0.007). Yoghurt products from Ecuador had lower salt (0.06 ± 0.02, *p* < 0.001), fibre (median (IQR)) (0 (0, 0), *p* = 0.003) and protein (mean ± SD) (3.4 ± 1.0, *p* = 0.006) but higher carbohydrate content (15 (13.3, 21.1), *p* = 0.018) than products in the UK, while products from Mexico had lower saturated fat (median (IQR)) (1.0 (0.9, 1.5), *p* = 0.01), salt (mean ± SD) (0.04 ± 0.02, *p* < 0.001) and protein content (3.0 ± 1.7, *p* < 0.001) (Table 3). Overall, one third of yoghurts were classified as “less healthy”. In comparison to the UK and the other Latin American countries, Ecuador had the highest proportion of less healthful products (Table 3). 

## 4. Discussion

The current study investigated whether differences exist in the nutritional quality, in particular, sugar content of REBCs and yoghurts marketed towards children in the UK, Mexico, Guatemala and Ecuador. We found that REBCs in the UK contain lower sugar and higher protein than those of Latin America, while yoghurts were generally similar across all countries in terms of nutritional content. Furthermore, a large proportion of REBCs and yoghurts in all countries were classified as “less healthy” according to the Ofcom NPM, but more products were classified as “less healthy” in Latin America. This is important as the market of these products is expanding in Latin America, and surveys show they are important contributors to the dietary intake of children [8,12,13,44]. Pervasive child-orientated marketing is associated with brand recognition and increased consumption; thus, stronger advocacy and political will is needed at a global level to reduce the promotion of less healthful foods to children [45]. A more recent concern in child-orientated food marketing is the use of health and nutrition claims in foods that are less healthful [19]. In this study, a higher proportion of products in the UK contained nutrient and health claims than those in Latin America. The use of nutrient and health claims in foods marketed towards children is another marketing strategy that needs regulation.

In the Latin American countries examined here, REBCs contained significantly higher sugar content than those of the UK. High sugar content of REBCs has been previously reported in several countries [9,19,26]. It is also known that REBCs targeting children are higher in total sugar than REBCs targeting adults [24]. However, a direct comparison of sugar content in REBCs between countries with different policies aiming to promote heathier food environments that include a sugar reduction policy is lacking. 

The significantly lower sugar content of UK REBCs observed in this study could be linked to the introduction of the voluntary sugar reduction programme; an 8.5% reduction in the sugar content of REBCs has been shown in the first progress report [7]. However, based on the classification previously used by our group and others for high sugar, the average sugar content of UK REBCs observed here still exceeds high sugar classifications of greater than 14.6 g/100g [19] by 10g. Thus, there is scope for further sugar reduction in the UK, especially as the programme is still ongoing. Food-specific reformulation is one of several actions in the sugar reduction programme, and it is important to promote all other aspects in cooperation with all stakeholders [46]. 

Food-specific sugar reduction policies have been implemented in some Latin American countries, although in Mexico and Ecuador, the existing sugar reduction policy has been mainly focused on sugar-sweetened beverages [5,6,47]. Other policies such as a new front-of-pack warning labelling system focused on energy, sugars, saturated fats and sodium have recently been approved by the Federal Executive in Mexico to encourage reformulation of food and beverage products. This policy would not have affected our results, because it was implemented after data collection [48]. In Ecuador, the implementation of the traffic light labelling system could have led to food reformulation, but little monitoring exists [49]. In Guatemala, where the food industry is less developed, there has only been one policy proposal, the Healthy Food Promotion Law 5504, which includes sugar, fat and sodium reduction, front of pack labelling and nutritional education, but this has not been approved by law yet [50]. Our findings highlight the need for more stringent sugar reduction policies that include a wider variety of foods relevant to children’s diets in Mexico, Guatemala and Ecuador.

The current study reports that a large proportion of child-orientated REBCs are less healthful; similar concerns have been reported by others [9,19,24,26]. The larger proportion of less healthful REBCs observed in the Latin American countries may be due to the lower protein and fibre combined with higher sugar content.

No differences in sugar content were observed in yoghurts among the countries examined. In a recent UK survey looking at the effect of the sugar reduction programme on yoghurts, a lower sugar content was observed in 61% of children’s yoghurts between 2016 and 2019. The average sugar value was 10 g/100g compared with 11.1 g/100g in our study; this small difference could be attributed to the different data collection times and the rapid turnover of products as reported by Moore et al. [51] Thus, it is possible that the changing nature of the UK market could have influenced our findings. Still, it is concerning that overall, more than one third of yoghurts were classified as “less healthy”. This may in part be explained by the higher levels of saturated fat and low fibre in these products [19]. A larger proportion of less healthful yoghurts were reported in Ecuador. This is likely due to the significantly lower amounts of protein, since sugar content was comparable to the other countries. The traffic light system was implemented in Ecuador in 2014 [49] with the aim to help consumer choice for healthier products. A sharp drop in yoghurt sales was reported in 2017 with red traffic lights due to high sugar content cited as an explanation for the reduction in sales [52], and this could have prompted reformulation of yoghurts.

This study was the first to compare the nutritional quality of REBCs and yoghurts between the UK and specific Latin American countries with differing policy and regulations. Data for each country were collected by two researchers who collected data individually to corroborate data entry and who were familiar with the locations and products in all four countries; this improved quality control of data reported and offered logistical benefits (organisation, manpower and costs). However, inter-coder reliability and coefficients of variation were not recorded, which is a limitation. Other limitations were the use of different methods of data collection (in store and online) and using nutrition information from food labels, which is not ideal for objectively reporting nutrient composition data. Furthermore, the analysis included products from just one large city within each country and therefore may not completely represent those foods available throughout each country. Some products may have been overlooked, as not all supermarkets were surveyed; thus, our study is not representative of all products available at the time of data collection. Finally, due to the cross-sectional nature of the study, we can only report descriptive data and associations at the specific time of data collection. 

## 5. Conclusions

The nutritional quality of REBCs and yoghurts in Latin America is different from that in the UK. The reformulation programme in the UK is likely to have contributed to the reduction of sugar content in REBCs. A large proportion of REBCs and yoghurts in this study are less healthful, but the proportion is larger in Latin America compared with that in the UK. Stakeholder willingness to implement strategies to improve the nutritional profile of foods marketed towards children is feasible as seen in the UK products, and these strategies could be adopted in countries where policies to improve the food environment are urgently needed. 

## Figures and Tables

**Table 1:** Description of types of marketing strategies and claims used on cereals and yoghurts. *n* (%).

	All	United Kingdom (*n* = 85)	Ecuador (*n* = 62)	Guatemala (*n* = 57)	Mexico (*n* = 58)
Marketing					
Cartoons	235 (89.7)	76 (89.4)	55 (88.7)	46 (80.7)	58 (100)
Toys	18 (6.9)	0	13 (21.0)	3 (5.3)	2 (3.4)
Promotion	11 (4.2)	0	1 (1.6)	8 (14.0)	2 (3.4)
Claims					
Health	61 (23.3)	37 (43.5)	6 (9.7)	9 (15.8)	9 (15.5)
Micronutrient	158 (60.3)	70 (82.4)	22 (35.5)	30 (52.6)	36 (62.1)
Fibre	31 (11.8)	12 (14.1)	0	10 (17.5)	9 (15.5)
No added sugars	16 (6.1)	5 (5.9)	0	1 (1.8)	10 (17.2)
Low fat and cholesterol	22 (8.4)	5 (5.9)	0	5 (8.8)	12 (20.7)
Other claims	50 (19.1)	33 (38.8)	10 (16.1)	7 (12.3)	0

**Table 2:** Comparison of energy and nutrient composition and nutrient profiling of ready to eat breakfast cereals (REBCs) in Guatemala, Mexico, Ecuador and the United Kingdom.

	All Countries	United Kingdom (*n* = 41)	Guatemala (*n* = 43)	Mexico (*n* = 31)	Ecuador (*n* = 39)
Energy/Nutrient					
Energy (KJ)	1650.3 ± 188.2	1681.5 ± 100.1	1595.8 ± 216.8	1594.2 ± 81.9	1713.9 ± 258.9
*p* value			0.251	0.267	1.0
Energy (Kcal)	392.0 ± 43.3	398.2 ± 25.6	381.1 ± 47.7	379.6 ± 21.2	407.8 ± 59.0
*p* value			0.391	0.386	1.0
Sugar (g)	30.7 ± 9.2	24.6 ± 6.41	31.5 ± 8.31	32.6 ± 7.6	34.6 ± 10.8
*p* value			0.001	0.001	<0.001
Fats (g) *	3.3 (1.0 to 5.0)	3.3 (2.0 to 11.0)	3.3 (1.7 to 5.0)	2.8 (0.8 to 3.7)	3.3 (0 to 5.0)
*p* value ^$^			1.0	1.0	1.0
Saturated fats (g) *	0.8 (0 to 1.8)	2.4 (1.7 to 4.4)	1.0 (0 to 1.8)	0.7 (0 to 1.7)	0 (0 to 1.7)
*p* value ^$^			0.434	0.158	0.002
Salt (g)	0.44 ± 0.2	0.51 ± 0.2	0.46 ± 0.2	0.45 ± 0.2	0.33 ± 0.2
*p* value			1.0	1.0	0.001
Fibre (g) *	3.2 (1.2 to 5.0)	4.3 (2.3 to 5.6)	2.7 (0 to 4.7)	2.3 (1.7 to 4.3)	0 (0 to 3.3)
*P* value ^$^			0.042	0.245	0.001
Carbohydrates (g) *	83.3 (76.4 to 86.7)	76.0 (72.0 to 83.8)	83.3 (79.6 to 86.5)	84.0 (80.0 to 86.6)	86.7 (82.5 to 86.7)
*p* value ^$^			0.095	0.005	<0.001
Proteins (g)	5.8 ± 1.9	6.8 ± 1.4	5.2 ± 1.6	5.2 ± 2.2	5.7 ± 2.1
*p* value			<0.001	0.001	0.033
Nutrient Profile ^+^					
“Healthy”	26 (16.9)	14 (34.1)	4 (9.3)	4 (12.9)	4 (10.3)
“Less healthy”	128 (83.1)	27 (65.9)	39 (90.7)	27 (87.1)	35 (89.7)

Values are reported as means ± Standard Deviation per 100 grams unless otherwise stated. *p* values represent comparisons between individual Latin American countries and the United Kingdom. * median (Inter quartile range). ^$^ *p* value: Kruskal–Wallis test. ^+^ *n* (%).

**Table 3:** Comparison of nutrient composition and nutrient profiling of yoghurt products from Guatemala, Mexico and Ecuador and the United Kingdom.

	All Countries	United Kingdom (*n* = 44)	Guatemala (*n* = 14)	Mexico (*n* = 26)	Ecuador (*n* = 23)
Energy/Nutrient					
Energy (KJ)	404.2 ± 133.3	401.4 ± 123.5	427.2 ± 78.1	342.9 ± 85.6	464.8 ± 187.8
*p* value			1.0	0.409	0.343
Energy (Kcal)	95.6 ± 32.5	95.2 ± 29.4	95.9 ± 22.5	81.9 ± 20.5	111.8 ± 46.1
*p* value			0.539	1.0	0.257
Sugar (g)	10.9 ± 3.2	11.1 ± 2.0	11.4 ± 3.9	10.7 ± 2.9	10.9 ± 4.8
*P* value			1.0	1.0	1.0
Fats (g) *	2.4 (1.8 to 2.8)	2.4 (1.9 to 2.8)	2.6 (2.3 to 2.9)	1.8 (1.6 to 2.6)	2.5 (2.2 to 4.7)
*p* value			1.0	0.161	0.745
Saturated fats (g) *	1.6 (1.0 to 1.9)	1.6 (1.2 to 1.9)	1.5 (1.0 to 1.7)	1.0 (0.9 to 1.5)	1.8 (1.6 to 3.5)
*p* value			1.0	0.013	0.128
Salt (g)	0.08 ± 0.06	0.13 ± 0.07	0.05 ± 0.02	0.04 ± 0.02	0.06 ± 0.02
*p* value			<0.001	<0.001	<0.001
Fibre (g) *	0 (0 to 0.28)	0.9 (0 to 0.4)	0.1 (0 to 0.3)	0 (0 to 0.1)	0 (0 to 0)
*p* value			1.0	0.078	0.003
Carbohydrates (g) *	13.3 (11.2 to 15.3)	12.0 (10.5 to 13.6)	16.3 (14.0 to 19.5)	12.3 (11.8 to 14.9)	15 (13.3 to 21.1)
*p* value			0.007	NS	0.018
Proteins (g)	3.8 ± 1.6	4.7 ± 1.5	3.6 ± 1.4	3.0 ± 1.7	3.4 ± 1.0
*p* value			0.098	<0.001	0.006
Nutrient Profile ^+^					
“Healthy”	72 (67.3)	33 (75.0)	13 (92.9)	17 (65.4)	9 (39.1)
“Less healthy”	35 (32.7)	11 (25.0)	1 (7.1)	9 (25.0)	14 (60.9)

Values are means ± Standard Deviation per 100 grams unless otherwise stated. NS Non significant, *p* values represent comparisons between individual Latin American countries and the United Kingdom. * median (Inter quartile range). *p* value: Kruskal–Wallis test. ^+^ *n* (%).
